# Integrating Nanotechnology in Neurosurgery, Neuroradiology, and Neuro-Oncology Practice—The Clinicians’ Perspective

**DOI:** 10.3389/fbioe.2022.801822

**Published:** 2022-02-09

**Authors:** Fred C. Lam, Fateme Salehi, Ekkehard M. Kasper

**Affiliations:** ^1^ Division of Neurosurgery, Hamilton Health Sciences, McMaster University Faculty of Health Sciences, Hamilton, ON, Canada; ^2^ Department of Radiology, Hamilton Health Sciences, McMaster University Faculty of Health Sciences, Hamilton, ON, Canada

**Keywords:** nanotechnology, neurosurgery, neuro-oncology, neuroradiology, neuro-imaging

## Introduction

Body scanners and operating microscopes have become standard of care for diagnostic imaging and operative planning in neurosurgical oncology. Recently, the preclinical development of novel nanoscale materials for use in enhancing imaging and visualization of brain tumors *in vivo* have led to translational platforms that offer clinicians the potential for improving surgical outcomes and tailoring personalized treatment regimens. In this piece written by three physician-scientists with over 30 years of combined expertise in neurosurgery, neuroradiology, neuro-oncology, and CNS nanotherapeutics, we provide our collective opinion regarding the emerging uses of nanotechnology in our respective subspecialties.

## Nanotechnology in Neuroradiology

Common neuroimaging modalities such as computed tomography (CT) and magnetic resonance imaging (MRI) provide anatomical details of the brain and spine. Both CT and MRI scans provide ∼25–100 μm resolution of neural structures (Kaviarasi et al., 2019). CT uses X-rays while MRI uses radiowaves and magnetic fields for image acquisition and iodine- or gadolinium (Gd)-based contrast dye agents, respectively, for further enhancement and delineation of lesions such as higher grade tumors, vascular lesions, or traumatic brain injuries that cause leakiness of the blood-brain barrier (BBB) (Martina et al., 2005; Bauer et al., 2014). Tracer-based imaging modalities such as positron emission spectroscopy (PET) and single-photon emission computerized tomography (SPECT) have limits of resolution between 2 and 10 mm (Moses, 2011; Bailey and Willowson, 2013) and rely on costly, injectable radioactive tracers to detect diseased cells.

Nanotechnology for neuroimaging largely remains in early stage preclinical development. Functionalized nanoparticles containing iron oxide or gold or quantum dots have been tested in mouse models of stroke and brain tumors, demonstrating enhanced visualization of tumor foci, thrombi, or infarcted brain tissues (reviewed in Kaviarasi et al., 2019) (Kaviarasi et al., 2019). In particular, high resolution magnetic particle imaging (MPI) utilizing superparamagnetic iron oxide nanoparticles to acquire quantitative three-dimensional, *in vivo* real-time imaging shows promise in the fields of vascular, tumor, and cell labeling and tracking (reviewed in Wu et al., 2019) (Wu et al., 2019). Furthermore, the use of artificial intelligence and machine learning to deconvolute neural networks with brain mapping will further compliment the uses of nanotechnology in molecular neuroimaging (Lui et al., 2020; Yao et al., 2020).

More recently, investigators have begun to use nanotechnology to improve the functionality and safety of Gd-based contrast agents for MRI. While Gd is the most frequently used metal ion for MRI scanning due to its high magnetic moment causing significant relaxation rates of nearby water protons, allowing for enhanced anatomical resolution and the visualization of higher-grade primary and metastatic brain tumors, the known renal toxicity of free Gd ions have led to the development of Gd-chelates such as Gd-DPTA (Magnevist, Schering AG), which rapidly clears the contrast agent through the kidneys to decrease the risk of kidney injury (Wang, 2011; Weidman et al., 2015; Russo et al., 2017). However, chelation reduces the relaxivity of Gd by decreasing the number of sites for water proton exchange, thus decreasing contrast enhancement. Several nanotechnologies have been shown to increase the relaxivity of Gd, including: 1) Geometric confinement of Magnevist, gadofullerenes, or gadonanotubes in porous silicon nanoparticles (Ananta et al., 2010); 2) Encapsulating Gd-DPTA in hyaluronic acid hydrogel nanoparticles to increase its hydrodenticity (Russo et al., 2017); and 3) Rigidification of Gd by constraining it as a tricyclic tetraazatriacetic Gd chelate (Port et al., 2006), all three methods of which have been shown to significantly increase proton relaxivity compared to that of clinically available Gd-based contrast agents.

Another emerging use of nanotechnology is in the field of molecular imaging, including PET and SPECT, which with their high sensitivity of detection, is being co-apted into multimodal imaging systems (PET/CT, SPECT/CT, PET/MRI) to allow for early cancer detection and/or personalized treatment algorithms (Weissleder, 2006; James and Gambhir, 2012; Goel et al., 2017). ^18^F-fluorodeoxyglucose (^18^F-FDG) is the most common radionuclide that is used as a PET tracer that is taken up by rapidly metabolizing cells such as malignant tumor cells, however its specificity for tumor cells is low–this has led to the development of nanomaterials that can be deployed as targeted molecular imaging probes simultaneously functionalized to target tumor cells and deliver therapeutic payloads (Weissleder, 2006; Hong et al., 2009). ^124^I-labeled gold nanostars have recently been shown to serve as a subcellular tracker for PET scanning in a preclinical mouse model of glioma with the potential for translational applications (Liu et al., 2019), while ^99m^Tc-labeled peptide targeting nanoprobes have shown promise in preclinical studies for glioma SPECT imaging (Zhao, 2016). The ability to combine these targeted molecular probes with multimodal imaging has the potential to improve the characterization of brain tumors at the time of diagnosis and throughout a patient’s course of treatment.

## Nanotechnology in Neurosurgery

The use of nanotechnology in neurosurgical procedures has gained much more maturity than in neuroradiology, with the application of nanomaterials across each subspecialty of neurosurgery. Nano-roughened titanium interbody cages that stimulate osteoblastic activation and osteointegration (Gittens et al., 2012); bioactive nanofiber scaffolds as carriers for recombinant human bone morphogenic protein (Lee et al., 2015), and polyethyl ether ketone nanocomposite polymers that assist in implant technologies for patients with osteoporotic bone (Li, 2012), are commonly used in spinal fusion surgeries (reviewed in Viswanathan et al., 2019) (Viswanathan et al., 2019).

Intra-operative fluorescence-guided surgery using the fluorescent pro-agent 5-aminolevulenic acid (5-ALA) and the fluorescent dye indocyanine green (ICG), both of which have demonstrated good penetrance across the blood-brain barrier (BBB), have shown promise in improving the extent of maximal safe surgical resection of brain tumors (Valdés et al., 2015; Teng et al., 2021). Similarly, a phase one safety trial of BLZ-100, a 36 amino acid synthetic peptide chlorotoxin derived from scorpion venom conjugated to ICG (Tozuleristide), has established dose-limiting toxicities and enhanced intra-operative fluorescence of newly diagnosed or recurrent adult gliomas (Patil et al., 2019). The increased prevalence of fluorescence-guided intraoperative surgical resection of brain tumors have been aided by the development of several intraoperative imaging systems, such as: The commonly available Leica OH6 microscope (Wetzlar, Germany) which is equipped with the FL800^TM^ module that has a high-sensitivity near-infrared (NIR) camera and 820–860 nm filter that detects NIR fluorescence and generates a black and white image that cannot be overlaid with visible light microscopy images (Cho, 2018; Cho et al., 2018); The Zeiss CONVIVO^®^ confocal laser endomicroscopy digital biopsy probe that allows for *in situ* visualization of fluorescein-labelled tissues and allows for seamless interfacing with their KINEVO^®^ 900 robotic visualization system combining fluorescent views of tissue microstructure with surgical views under the operating microscope (Zeiss, Oberkochen, Germany); And finally, the FDA-approved VisionSense Iridium^TM^ handheld NIR exoscope (VisionSense, Philadelphia, PA) which is less commonly used in neurosurgery but more widely used in plastic surgery and reconstructive general surgery procedures, and allows for overlay of fluorescence and white light images in real time (AV, 2016; Cho et al., 2018). These intra-operative nanoscale imaging materials and fluorescence imaging systems have the potential to maximize the extent of safe surgical resection of highly malignant brain tumors with hopes of prolonging patient survival.

Nanomaterials that promote osteoblastic stem cell growth in intervertebral cages made out of titanium or polyetheretherketone (PEEK) have dramatically improved the fusion rates in spine surgery (Girasole et al., 2013). Nanofibrous poly (D,L-lactide-co-e-caprolactone) balloons have been used in verteplasty procedures to fill compressed fractured vertebra (Sun et al., 2013). Finally, experimental collagen scaffolds of collagen-binding brain-derived neurotrophic factor have been shown therapeutic promise in a canine model of spinal cord injury (Andrychowski et al., 2013).

## Nanotechnology in Neuro-Oncology

Currently, the food and drug administration (FDA) has approved albumin-bound paclitaxel and pegylated liposomal doxorubicin for used as systemic anti-cancer therapies. These nanomedicines do not cross the BBB and therefore are not appropriate for use in the neuro-oncology space. A variety of nanoparticles have been developed in the preclinical testing phases that have shown promising results to be able to circumvent the BBB for delivery of small molecule inhibitors, chemotherapies, antibody-drug conjugates, and gene therapies in murine models of gliomas (Glaser et al., 2017; Grabowska et al., 2019). We recently reported enhanced safety and efficacy of nanoliposomes functionalized with transferrin, which aids in receptor-mediated endocytosis across the BBB and targeting to glioma cells via the expression of transferrin receptors on the surfaces of both endothelial and glioma cells, in the delivery of combination anti-cancer therapies to murine models of gliomas (Lam et al., 2018). However, all these nanoscale materials are still restricted to preclinical studies and have yet to translate into the clinical setting for the treatment of patients with brain tumors.

Recent advances in photon beam radiation therapy (RT) techniques for the treatment of brain tumors have allowed for the ability to deliver high doses of radiation precisely to different regions of the brain. Treatment planning algorithms such as intensity- or volume-modulated radiotherapy/arc therapy (Sheu et al., 2019), when applied to techniques such as stereotactic radiosurgery and fractionated RT (Scaringi et al., 2018), have allowed radiation oncologists to deliver highly conformed doses of radiation to tumors while sparing adjacent normal neurovasculature with promising preliminary outcomes in maintaining high local control rates (Ruggieri et al., 2018). However, RT treatments are associated with acute and late central nervous system toxicities, including headaches, vomiting, motor/sensory neuropathy, cognitive deficits, and seizures (Brown et al., 2016; Chen et al., 2017). One emerging modality to avoid these toxicities is proton beam therapy, which uses positively charged elementary particles to deposit a sharp peak of energy (the Bragg Peak) to the target tumor volume with minimal exit dose to decrease the rates of acute and late toxicities and increases the therapeutic ratio of RT (Sherman et al., 2016; Indelicato et al., 2019; Weber et al., 2020). However, it has also been postulated that the high transfer of energy at the distal range of the beam may be also cause toxicities (Peeler et al., 2016; Haas-Kogan et al., 2018). To further increase the therapeutic window and minimize dose-related toxicities, the development of nanoscintillators, down-conversion NPs that absorb x-rays and emit a wide-range of photons, have been used as photosensitizers in photodynamic therapy to produce high amounts of reactive oxygen species when delivered locally into tumor tissues, creating cytotoxicity and tumor cell killing (Castano et al., 2006; Celli et al., 2010). These include rare-earth lanthanum/iron/ceramide-based composite NPs that have shown promise in reducing radiation treatment-induced toxicities and improving therapeutic benefits in preclinical models of brain tumors (Bulin et al., 2020).

## Current Limitations of Nanotechnology for Use in the Human Central Nervous System

Limitations to successful translation of most benchtop nanotechnologies into the clinic have been due to the potential systemic toxicities of NPs. FDA-approved pegylated liposomal doxorubicin (Doxil^®^) has increased systemic circulation times, reduced cardiotoxicity, and demonstrated similar efficacy compared to conventional doxorubicin for the treatment of metastatic breast cancer (O’Brien et al., 2004). Compared to other types of nanotechnologies composed of organic, inorganic, or metal components, liposomes and other lipid-based NPs appear to have the lowest toxicity profiles *in vivo* (Puri et al., 2009). However, patients taking Doxil^®^ have reported increased incidences of hand-foot syndrome and developed cutaneous squamous cell carcinoma after repeated use (Anselmo and Mitragotri, 2019; Pease et al., 2019). A recent systematic review of 14 randomized clinical trials comparing the efficacy of liposomal encapsulated cytotoxic therapies to equivalent conventional formulations did not show improved efficacy in humans despite showing significantly increased survival in tumor-bearing mice (Petersen et al., 2016). This review exemplifies deficiencies in understanding the pharmacokinetics and pharmacodynamics of nanomedicines in humans and the limitations for clinical use. Little is known regarding the long-term effects of NP deposition in human organs, however animal studies have shown increased cellular oxidative stress in different organs (Dick et al., 2003; Donaldson, 2004). Other purported mechansims of toxicity include generation of DNA damage, protein structural and functional modifications, and disruption of membrane integrity (reviewed in Najahi-Missaoui et al., 2021) (Najahi-Missaoui et al., 2020). Furthermore, most preclinical studies have manufactured NPs under non-GLP (Good Laboratory Practices)/non-GMP (Good Manufacturing Practice) laboratory environments in relatively small batches, thus without knowledge of how large scale-up production of NPs can affect the quality and consistency of the final product, it becomes difficult to assess the safety and efficacy of these nanomedicines in humans.

**FIGURE 1:**
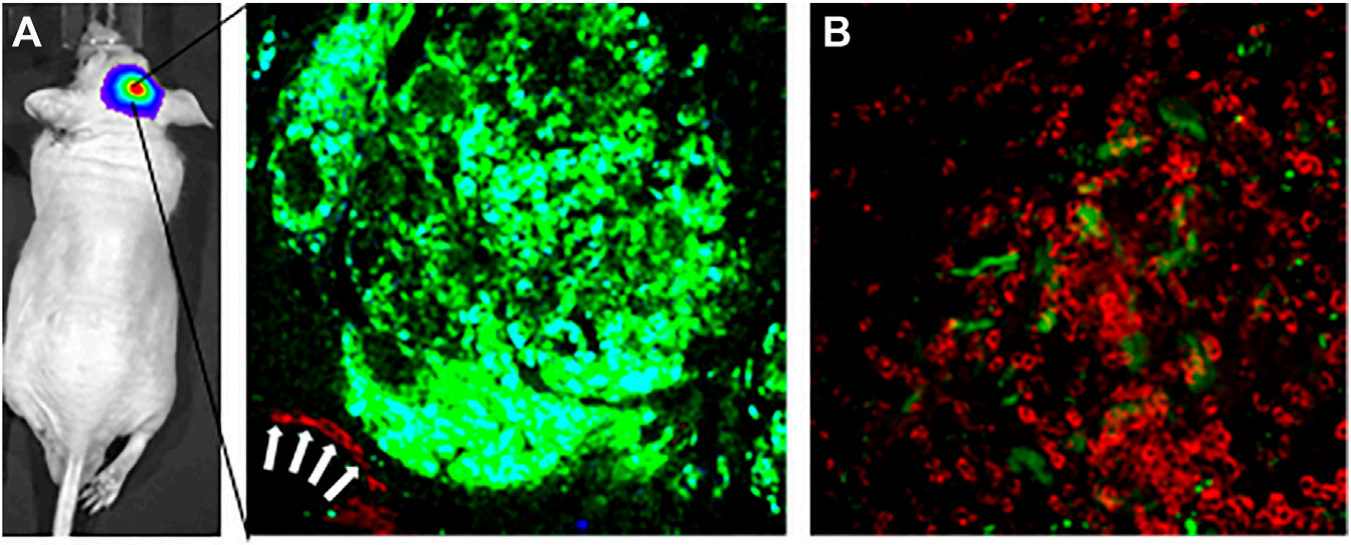
Intravital multiphoton imaging demonstrates delivery of fluorescent tumor-targeting liposomal nanoparticles across the blood-brain barrier to a glioma brain tumor in an intracranial orthotopic xenograft mouse model of glioma. **(A)** Left panel-intravital multiphoton image through a cranical window of a GFP-expressing glioma tumor. White arrows show Cy5.5-conjugated liposomal nanoparticles coursing throught a blood vessel adjacent to the tumor. Imaged immediately after tail vein injection of nanoparticles. **(B)** Intravital mutiphoton image taken 24 h following tail vein injection showing accumulation of Cy5.5-conjugated liposomal nanoparticles on the surface of the brain tumor. Accumulation Cy5.5 signal in cells with a bulls-eye center suggestive of uptake of nanoparticles by resident brain microglia.

Preclinical studies have also shown mechanisms of neurotoxicity associated with nanomaterials. Interactions of nanomaterials with glial cells and neurons have led to oxidative burst activity in microglia (Long et al., 2006; Ze et al., 2013; Shrivastava et al., 2014), inflammation due to the release of cytokines and tumor necrosis factor-α by microglia (Li et al., 2009; Ze et al., 2014), DNA damage (Golbamaki et al., 2015; Ren et al., 2016), and apoptosis (Márquez-Ramírez et al., 2012; Ganguly et al., 2018). Our experience with glioma-targeted liposomal NP delivery of cytotoxic therapies across the BBB in mouse models of glioma has demonstrated successful accumulation of transferrin-functionalized NPs on the surface of glioma tumors (Lam et al., 2018). Intravital multiphoton imaging through a cranial window into the brains of these glioma bearing mice (Figure 1A, glioma tumor in green) immediately after systemic injection of fluorescence-conjugated liposomal NPs demonstrates the presence of NPs flowing through an adjacent tumor vessel (Figure 1A, white arrows pointing to liposomal NPs in red). Twenty-four hours after systemic injection, imaging demonstrated accumulation of NPs on the surface of the tumor (Figure 1B, tumor signal in green, liposomal NPs in red). Red fluorescence signal is seen in cells surrounding the tumor with a bulls-eye appearance, likely suggestive of scavenger uptake of NPs by resident microglia, which would serve as a mechanism for the eventual degradation and clearance of NPs from the CNS milieu (Figure 1).

## New Horizons

Despite the potential for nanoscale applications to revolutionize the fields of neuroradiology, neuro-oncology, and neurosurgery, the largely impenetrable BBB remains to be a major hindrance in the effective delivery of payload into the CNS. To circumvent the BBB, researchers have exploited both intranasal and intrathecal delivery routes for direct delivery into the CNS. The nasal mucosa provides a direct route into the brain via the olfactory epithelium through the cribriform plate of the skull base (Tashima, 2020). Most intranasal medications have been marketed for local or systemic delivery to the nose or the upper respiratory tract for indications such as allergic rhinitis. More recently, micellar, liposomal, and chitosan NPs have been used to deliver insulin intranasally into the brain for the treatment of Alzheimer’s disease (reviewed in Tashima et al., 2020), paving the road for the possibility of using insulin as a backbone conjugate linker to carry other small molecules intranasally into the CNS space to treat other CNS disorders (Tashima, 2020).

Intrathecal delivery of therapeutics can also circumvent systemic barriers to CNS drug delivery to achieve high concentrations of drugs in the CSF while averting systemic exposure and have shown promise in both the clinical and preclinical space for the treatment of intraventricular tumors and leptomeningeal disease (Sandberg et al., 2012; Bottros and Christo, 2014; Chen et al., 2015; Sandberg et al., 2015). A recent study characterizing the biodistribution of 100 nm fluorescent-conjugated PEGylated NPs delivered into the cisterna magna of healthy mice found that these NPs were evenly distributed throughout the subarachnoid space along the brain and spinal cord with retention in the leptomeninges for up to 3 weeks–however, there was minimal penetration into the brain parenchyma (Householder et al., 2019). This points towards the selective use of intrathecal delivery for the treatment of diseases with an affinity for the meninges, such as leptomeningeal carcinomatosis, for which implantable intraventricular devices such as the Neuroinfuse^TM^ multicatheter infusion device (Renishaw, United Kingdom) are currently in human clinical trials (Chen et al., 2015), or the need to further functionalize nanoparticles to allow for cell type-specific delivery within the brain.

Finally, the field of theranostics–combining therapeutic and diagnostic capabilities into a single NPs delivery system have gradually come into the forefront of personalized medicine, potentially offering time and cost savings while improving patient outcomes (Lammers et al., 2010; Wang et al., 2014). Multifunctionalized theranostic NPs that contain imaging agents such as iron oxide, chemotherapeutic agents such as doxorubicin, and tumor cell-targeting moieties such as folate, have the ability to allow for real-time imaging and tracking of therapeutic responses for the treatment of brain tumors (reviewed in d’Angelo et al., 2019) (d’Angelo et al., 2019). Superparamagnetic iron oxide and doxorubicin loaded into chitosan NPs have the ability to be internalized by C6 glioma cells, demonstrating a decline in T2 relaxation times *in vitro* during MRI scanning, paving the road for further preclinical testing of theranostic NPs for the treatment of brain tumors (Gholami et al., 2019). The ability to deliver multimodal NPs using novel routes of delivery into the CNS space could further the field of personalized medicine for the treatment of brain tumors.

## Discussion

From a neurosurgeon’s perspective, we often implant foreign materials into the brain, including Ommaya reservoirs (Magill et al., 2020), external ventricular catheters (Dey et al., 2015), and deep brain stimulator electrodes (Engel et al., 2018), composed of inert soft plastics, as well biodegradable wafers impregnated with the cytotoxic agent carmustine (Gliadel^®^) into the surgical resection cavity for the treatment of gliomas (Chowdhary et al., 2015). Some reported side-effects of these implantable devices include migration within the brain leading to inadvertent hemorrhage and neurologic injury, brain swelling due to foreign body reactions, seizures, and scarring. Extrapolating our experience using macromolecular intracranial implants, there is a need to design early phase clinical trials to thoroughly determine the safety of all novel nanomaterials in the CNS leading into larger phase II/III randomized trials to assess the efficacy of these materials for their intended CNS applications.

## Conclusion

Nanomaterials for use in CNS applications is a rapidly emerging space with much potential for improving the care of patients with neurological and neurosurgical issues. There remains a large translational gap between benchtop to clinical studies but the increasing involvement of clinician scientists with an interest in nanomaterials research will undoubtedly narrow that gap.

## References

[B1] Ananta J. S. Godin B. Sethi R. Moriggi L. Liu X. Serda R. E. (2010). Geometrical Confinement of Gadolinium-Based Contrast Agents in Nanoporous Particles Enhances T1 Contrast. Nat. Nanotech 5 (11), 815–821. 10.1038/nnano.2010.203 PMC2974055 20972435

[B2] Andrychowski J. Frontczak-Baniewicz M. Sulejczak D. Kowalczyk T. Chmielewski T. Czernicki Z. (2013). Original Article Nanofiber Nets in Prevention of Cicatrisation in Spinal Procedures. Experimental Study. fn 2 (2), 147–157. 10.5114/fn.2013.35958 23821387

[B3] Anselmo A. C. Mitragotri S. (2019). Nanoparticles in the Clinic: An Update. Bioeng. Transl Med. 4 (3), e10143. 10.1002/btm2.10143 31572799 PMC6764803

[B4] Av D. S., (2016). Review of Fluorescence Guided Surgery Systems: Identification of Key Performance Capabilities beyond Indocyanine green Imaging. J. Biomed. Opt. 21 (8), 80901. 27533438 10.1117/1.JBO.21.8.080901 PMC4985715

[B5] Bailey D. L. Willowson K. P. (2013). An Evidence-Based Review of Quantitative SPECT Imaging and Potential Clinical Applications. J. Nucl. Med. 54 (1), 83–89. 10.2967/jnumed.112.111476 23283563

[B6] Bauer A. Q. Kraft A. W. Wright P. W. Snyder A. Z. Lee J.-M. Culver J. P. (2014). Optical Imaging of Disrupted Functional Connectivity Following Ischemic Stroke in Mice. Neuroimage 99, 388–401. 10.1016/j.neuroimage.2014.05.051 24862071 PMC4332714

[B7] Bottros M. M. Christo P. J. (2014). Current Perspectives on Intrathecal Drug Delivery. J. Pain Res. 7, 615–626. 10.2147/JPR.S37591 25395870 PMC4227625

[B8] Brown P. D. Jaeckle K. Ballman K. V. Farace E. Cerhan J. H. Anderson S. K. (2016). Effect of Radiosurgery Alone vs Radiosurgery with Whole Brain Radiation Therapy on Cognitive Function in Patients with 1 to 3 Brain Metastases. JAMA 316 (4), 401–409. 10.1001/jama.2016.9839 27458945 PMC5313044

[B9] Bulin A. L. Broekgaarden M. Chaput F. Baisamy V. Garrevoet J. Busser B. (2020). Radiation Dose‐Enhancement Is a Potent Radiotherapeutic Effect of Rare‐Earth Composite Nanoscintillators in Preclinical Models of Glioblastoma. Adv. Sci. 7 (20), 2001675. 10.1002/advs.202001675 PMC7578894 33101867

[B10] Castano A. P. Mroz P. Hamblin M. R. (2006). Photodynamic Therapy and Anti-tumour Immunity. Nat. Rev. Cancer 6 (7), 535–545. 10.1038/nrc1894 16794636 PMC2933780

[B11] Celli J. P. Spring B. Q. Rizvi I. Evans C. L. Samkoe K. S. Verma S. (2010). Imaging and Photodynamic Therapy: Mechanisms, Monitoring, and Optimization. Chem. Rev. 110 (5), 2795–2838. 10.1021/cr900300p 20353192 PMC2896821

[B12] Chen L. Shen C. Redmond K. J. Page B. R. Kummerlowe M. Mcnutt T. (2017). Use of Stereotactic Radiosurgery in Elderly and Very Elderly Patients with Brain Metastases to Limit Toxicity Associated with Whole Brain Radiation Therapy. Int. J. Radiat. Oncology*Biology*Physics 98 (4), 939–947. 10.1016/j.ijrobp.2017.02.031 28602418

[B13] Chen T. C. Napolitano G. R. Adell F. Schönthal A. H. Shachar Y. (2015). Development of the Metronomic Biofeedback Pump for Leptomeningeal Carcinomatosis: Technical Note. Jns 123 (2), 362–372. 10.3171/2014.10.jns14343 25955873

[B14] Cho S. S. Zeh R. Pierce J. T. Salinas R. Singhal S. Lee J. Y. K. (2018). Comparison of Near-Infrared Imaging Camera Systems for Intracranial Tumor Detection. Mol. Imaging Biol. 20 (2), 213–220. 10.1007/s11307-017-1107-5 28741043 PMC11145178

[B64] Cho (2018). Mol Imaging Biol 20, 213–220. 28741043 10.1007/s11307-017-1107-5 PMC11145178

[B15] Chowdhary S. A. Ryken T. Newton H. B. (2015). Survival Outcomes and Safety of Carmustine Wafers in the Treatment of High-Grade Gliomas: a Meta-Analysis. J. Neurooncol. 122 (2), 367–382. 10.1007/s11060-015-1724-2 25630625 PMC4368843

[B16] d'Angelo M. Castelli V. Benedetti E. Antonosante A. Catanesi M. Dominguez-Benot R. (2019). Theranostic Nanomedicine for Malignant Gliomas. Front. Bioeng. Biotechnol. 7, 325. 10.3389/fbioe.2019.00325 31799246 PMC6868071

[B17] Dey M. Stadnik A. Riad F. Zhang L. McBee N. Kase C. (2015). Bleeding and Infection with External Ventricular Drainage. Neurosurgery 76 (3), 291–301. ; discussion 301. 10.1227/neu.0000000000000624 25635887 PMC4333009

[B18] Dick C. A. J. Brown D. M. Donaldson K. Stone V. (2003). The Role of Free Radicals in the Toxic and Inflammatory Effects of Four Different Ultrafine Particle Types. Inhalation Toxicol. 15 (1), 39–52. 10.1080/08958370304454 12476359

[B19] Donaldson K., (2004). Nanotoxicology. Occup. Environ. Med. 61 (9), 727–728. 10.1136/oem.2004.013243 15317911 PMC1763673

[B20] Engel K. Huckhagel T. Gulberti A. Pötter-Nerger M. Vettorazzi E. Hidding U. (2018). Towards Unambiguous Reporting of Complications Related to Deep Brain Stimulation Surgery: A Retrospective Single-center Analysis and Systematic Review of the Literature. PLoS One 13 (8), e0198529. 10.1371/journal.pone.0198529 30071021 PMC6071984

[B21] Ganguly P. Breen A. Pillai S. C. (2018). Toxicity of Nanomaterials: Exposure, Pathways, Assessment, and Recent Advances. ACS Biomater. Sci. Eng. 4 (7), 2237–2275. 10.1021/acsbiomaterials.8b00068 33435097

[B22] Gholami L. Tafaghodi M. Abbasi B. Daroudi M. Kazemi Oskuee R. (2019). Preparation of Superparamagnetic Iron Oxide/doxorubicin Loaded Chitosan Nanoparticles as a Promising Glioblastoma Theranostic Tool. J. Cel Physiol 234 (2), 1547–1559. 10.1002/jcp.27019 30145790

[B23] Girasole G. Muro G. Mintz A. Chertoff J. (2013). Transforaminal Lumbar Interbody Fusion Rates in Patients Using a Novel Titanium Implant and Demineralized Cancellous Allograft Bone Sponge. Int. J. Spine Surg. 7 (1), e95–e100. 10.1016/j.ijsp.2013.08.001 25580378 PMC4288454

[B24] Gittens R. A. Olivares-Navarrete R. McLachlan T. Cai Y. Hyzy S. L. Schneider J. M. (2012). Differential Responses of Osteoblast Lineage Cells to Nanotopographically-Modified, Microroughened Titanium-Aluminum-Vanadium alloy Surfaces. Biomaterials 33 (35), 8986–8994. 10.1016/j.biomaterials.2012.08.059 22989383 PMC3618458

[B25] Glaser T. Han I. Wu L. Zeng X. (2017). Targeted Nanotechnology in Glioblastoma Multiforme. Front. Pharmacol. 8, 166. 10.3389/fphar.2017.00166 28408882 PMC5374154

[B26] Goel S. England C. G. Chen F. Cai W. (2017). Positron Emission Tomography and Nanotechnology: A Dynamic Duo for Cancer Theranostics. Adv. Drug Deliv. Rev. 113, 157–176. 10.1016/j.addr.2016.08.001 27521055 PMC5299094

[B27] Golbamaki N. Rasulev B. Cassano A. Marchese Robinson R. L. Benfenati E. Leszczynski J. (2015). Genotoxicity of Metal Oxide Nanomaterials: Review of Recent Data and Discussion of Possible Mechanisms. Nanoscale 7 (6), 2154–2198. 10.1039/c4nr06670g 25580680

[B28] Grabowska M. Grześkowiak B. F. Szutkowski K. Wawrzyniak D. Głodowicz P. Barciszewski J. (2019). Nano-mediated Delivery of Double-Stranded RNA for Gene Therapy of Glioblastoma Multiforme. PLoS One 14 (3), e0213852. 10.1371/journal.pone.0213852 30889203 PMC6424419

[B29] Haas-Kogan D. Indelicato D. Paganetti H. Esiashvili N. Mahajan A. Yock T. (2018). National Cancer Institute Workshop on Proton Therapy for Children: Considerations Regarding Brainstem Injury. Int. J. Radiat. Oncology*Biology*Physics 101 (1), 152–168. 10.1016/j.ijrobp.2018.01.013 PMC5903576 29619963

[B30] Hong H. Zhang Y. Sun J. Cai W. (2009). Molecular Imaging and Therapy of Cancer with Radiolabeled Nanoparticles. Nano Today 4 (5), 399–413. 10.1016/j.nantod.2009.07.001 20161038 PMC2753977

[B31] Householder K. T. Dharmaraj S. Sandberg D. I. Wechsler-Reya R. J. Sirianni R. W. (2019). Fate of Nanoparticles in the central Nervous System after Intrathecal Injection in Healthy Mice. Sci. Rep. 9 (1), 12587. 10.1038/s41598-019-49028-w 31467368 PMC6715675

[B32] Indelicato D. J. Rotondo R. L. Uezono H. Sandler E. S. Aldana P. R. Ranalli N. J. (2019). Outcomes Following Proton Therapy for Pediatric Low-Grade Glioma. Int. J. Radiat. Oncology*Biology*Physics 104 (1), 149–156. 10.1016/j.ijrobp.2019.01.078 30684665

[B33] James M. L. Gambhir S. S. (2012). A Molecular Imaging Primer: Modalities, Imaging Agents, and Applications. Physiol. Rev. 92 (2), 897–965. 10.1152/physrev.00049.2010 22535898

[B34] Kaviarasi S. Yuba E. Harada A. Krishnan U. M. (2019). Emerging Paradigms in Nanotechnology for Imaging and Treatment of Cerebral Ischemia. J. Controlled Release 300, 22–45. 10.1016/j.jconrel.2019.02.031 30802476

[B35] Lam F. C. Morton S. W. Wyckoff J. Vu Han T.-L. Hwang M. K. Maffa A. (2018). Enhanced Efficacy of Combined Temozolomide and Bromodomain Inhibitor Therapy for Gliomas Using Targeted Nanoparticles. Nat. Commun. 9 (1), 1991. 10.1038/s41467-018-04315-4 29777137 PMC5959860

[B36] Lammers T. Kiessling F. Hennink W. E. Storm G. (2010). Nanotheranostics and Image-Guided Drug Delivery: Current Concepts and Future Directions. Mol. Pharmaceutics 7 (6), 1899–1912. 10.1021/mp100228v 20822168

[B37] Lee S. S. Hsu E. L. Mendoza M. Ghodasra J. Nickoli M. S. Ashtekar A. (2015). Gel Scaffolds of BMP-2-Binding Peptide Amphiphile Nanofibers for Spinal Arthrodesis. Adv. Healthc. Mater. 4 (1), 131–141. 10.1002/adhm.201400129 24753455 PMC4206675

[B38] Li K., (2012). Sintered Hydroxyapatite/polyetheretherketone Nanocomposites: Mechanical Behavior and Biocompatibility. Adv. Eng. Mater. 14, 155–165. 10.1002/adem.201080145

[B39] Li X.-b. Zheng H. Zhang Z.-r. Li M. Huang Z.-y. Schluesener H. J. (2009). Glia Activation Induced by Peripheral Administration of Aluminum Oxide Nanoparticles in Rat Brains. Nanomedicine: Nanotechnology, Biol. Med. 5 (4), 473–479. 10.1016/j.nano.2009.01.013 19523415

[B40] Liu Y. Carpenter A. B. Pirozzi C. J. Yuan H. Waitkus M. S. Zhou Z. (2019). Non-invasive Sensitive Brain Tumor Detection Using Dual-Modality Bioimaging Nanoprobe. Nanotechnology 30 (27), 275101. 10.1088/1361-6528/ab0e9c 30856613 PMC6948110

[B41] Long T. C. Saleh N. Tilton R. D. Lowry G. V. Veronesi B. (2006). Titanium Dioxide (P25) Produces Reactive Oxygen Species in Immortalized Brain Microglia (BV2): Implications for Nanoparticle Neurotoxicity. Environ. Sci. Technol. 40 (14), 4346–4352. 10.1021/es060589n 16903269

[B42] Lui Y. W. Chang P. D. Zaharchuk G. Barboriak D. P. Flanders A. E. Wintermark M. (2020). Artificial Intelligence in Neuroradiology: Current Status and Future Directions. AJNR Am. J. Neuroradiol 41 (8), E52–E59. 10.3174/ajnr.A6681 32732276 PMC7658873

[B43] Magill S. T. Choy W. Nguyen M. P. McDermott M. W. (2020). Ommaya Reservoir Insertion: A Technical Note. Cureus 12 (4), e7731. 10.7759/cureus.7731 32432009 PMC7234073

[B44] Márquez-Ramírez S. G. Delgado-Buenrostro N. L. Chirino Y. I. Iglesias G. G. López-Marure R. (2012). Titanium Dioxide Nanoparticles Inhibit Proliferation and Induce Morphological Changes and Apoptosis in Glial Cells. Toxicology 302 (2-3), 146–156. 10.1016/j.tox.2012.09.005 23044362

[B45] Martina A. D. Meyer-Wiethe K. Allémann E. Seidel G. (2005). Ultrasound Contrast Agents for Brain Perfusion Imaging and Ischemic Stroke Therapy. J. Neuroimaging 15 (3), 217–232. 10.1111/j.1552-6569.2005.tb00314.x 15951404

[B46] Moses W. W. (2011). Fundamental Limits of Spatial Resolution in PET. Nucl. Instr. Methods Phys. Res. Section A: Acc. Spectrometers, Detectors Associated Equipment 648 (Suppl. 1), S236–S240. 10.1016/j.nima.2010.11.092 PMC3144741 21804677

[B47] Najahi-Missaoui W. Arnold R. D. Cummings B. S. (2020). Safe Nanoparticles: Are We There yet. Int. J. Mol. Sci. 22 (1). 10.3390/ijms22010385 PMC7794803 33396561

[B48] O'Brien M. E. Wigler N. Inbar M. Rosso R. Grischke E. Santoro A. (2004). Reduced Cardiotoxicity and Comparable Efficacy in a Phase III Trial of Pegylated Liposomal Doxorubicin HCl (CAELYX/Doxil) versus Conventional Doxorubicin for First-Line Treatment of Metastatic Breast Cancer. Ann. Oncol. 15 (3), 440–449. 10.1093/annonc/mdh097 14998846

[B49] Patil C. G. Walker D. G. Miller D. M. Butte P. Morrison B. Kittle D. S. (2019). Phase 1 Safety, Pharmacokinetics, and Fluorescence Imaging Study of Tozuleristide (BLZ-100) in Adults with Newly Diagnosed or Recurrent Gliomas. Neurosurg. 85 (4), E641–E649. 10.1093/neuros/nyz125 31069381

[B50] Pease D. F. Peterson B. A. Gilles S. Hordinsky M. K. Bohjanen K. A. Skubitz K. M. (2019). Development of Cutaneous Squamous Cell Carcinoma after Prolonged Exposure to Pegylated Liposomal Doxorubicin and Hand-Foot Syndrome: a Newly Recognized Toxicity. Cancer Chemother. Pharmacol. 84 (1), 217–221. 10.1007/s00280-019-03849-8 31041511

[B51] Peeler C. R. Mirkovic D. Titt U. Blanchard P. Gunther J. R. Mahajan A. (2016). Clinical Evidence of Variable Proton Biological Effectiveness in Pediatric Patients Treated for Ependymoma. Radiother. Oncol. 121 (3), 395–401. 10.1016/j.radonc.2016.11.001 27863964 PMC5450501

[B52] Petersen G. H. Alzghari S. K. Chee W. Sankari S. S. La-Beck N. M. (2016). Meta-analysis of Clinical and Preclinical Studies Comparing the Anticancer Efficacy of Liposomal versus Conventional Non-liposomal Doxorubicin. J. Controlled Release 232, 255–264. 10.1016/j.jconrel.2016.04.028 27108612

[B53] Port M. Raynal I. Vander Elst L. Muller R. N. Dioury F. Ferroud C. (2006). Impact of Rigidification on Relaxometric Properties of a Tricyclic Tetraazatriacetic Gadolinium Chelate. Contrast Media Mol. Imaging 1 (3), 121–127. 10.1002/cmmi.99 17193688

[B54] Puri A. Loomis K. Smith B. Lee J.-H. Yavlovich A. Heldman E. (2009). Lipid-based Nanoparticles as Pharmaceutical Drug Carriers: from Concepts to Clinic. Crit. Rev. Ther. Drug Carrier Syst. 26 (6), 523–580. 10.1615/critrevtherdrugcarriersyst.v26.i6.10 20402623 PMC2885142

[B55] Ren C. Hu X. Li X. Zhou Q. (2016). Ultra-trace Graphene Oxide in a Water Environment Triggers Parkinson's Disease-like Symptoms and Metabolic Disturbance in Zebrafish Larvae. Biomaterials 93, 83–94. 10.1016/j.biomaterials.2016.03.036 27085073

[B56] Ruggieri R. Naccarato S. Mazzola R. Ricchetti F. Corradini S. Fiorentino A. (2018). Linac-based VMAT Radiosurgery for Multiple Brain Lesions: Comparison between a Conventional Multi-Isocenter Approach and a New Dedicated Mono-Isocenter Technique. Radiat. Oncol. 13 (1), 38. 10.1186/s13014-018-0985-2 29506539 PMC5836328

[B57] Russo M. Ponsiglione A. M. Forte E. Netti P. A. Torino E. (2017). Hydrodenticity to Enhance Relaxivity of Gadolinium-DTPA within Crosslinked Hyaluronic Acid Nanoparticles. Nanomedicine 12 (18), 2199–2210. 10.2217/nnm-2017-0098 28816102

[B58] Sandberg D. I. Peet M. M. Johnson M. D. Cole P. Koru-Sengul T. Luqman A. W. (2012). Chemotherapy Administration Directly into the Fourth Ventricle in a Nonhuman Primate Model. Ped 9 (5), 530–541. 10.3171/2012.1.peds11410 22546032

[B59] Sandberg D. I. Rytting M. Zaky W. Kerr M. Ketonen L. Kundu U. (2015). Methotrexate Administration Directly into the Fourth Ventricle in Children with Malignant Fourth Ventricular Brain Tumors: a Pilot Clinical Trial. J. Neurooncol. 125 (1), 133–141. 10.1007/s11060-015-1878-y 26255071 PMC4592494

[B60] Scaringi C. Agolli L. Minniti G. (2018). Technical Advances in Radiation Therapy for Brain Tumors. Anticancer Res. 38 (11), 6041–6045. 10.21873/anticanres.12954 30396918

[B61] Sherman J. C. Colvin M. K. Mancuso S. M. Batchelor T. T. Oh K. S. Loeffler J. S. (2016). Neurocognitive Effects of Proton Radiation Therapy in Adults with Low-Grade Glioma. J. Neurooncol. 126 (1), 157–164. 10.1007/s11060-015-1952-5 26498439 PMC12895352

[B62] Sheu T. Briere T. M. Olanrewaju A. M. McAleer M. F. (2019). Intensity Modulated Radiation Therapy versus Volumetric Arc Radiation Therapy in the Treatment of Glioblastoma-Does Clinical Benefit Follow Dosimetric Advantage. Adv. Radiat. Oncol. 4 (1), 50–56. 10.1016/j.adro.2018.09.010 30706010 PMC6349632

[B63] Shrivastava R. Raza S. Yadav A. Kushwaha P. Flora S. J. S. (2014). Effects of Sub-acute Exposure to TiO2, ZnO and Al2O3nanoparticles on Oxidative Stress and Histological Changes in Mouse Liver and Brain. Drug Chem. Toxicol. 37 (3), 336–347. 10.3109/01480545.2013.866134 24344737

[B65] Sun G. Wei D. Liu X. Chen Y. Li M. He D. (2013). Novel Biodegradable Electrospun Nanofibrous P(DLLA-CL) Balloons for the Treatment of Vertebral Compression Fractures. Nanomedicine: Nanotechnology, Biol. Med. 9 (6), 829–838. 10.1016/j.nano.2012.12.003 23318398

[B66] Tashima T. (2020). Shortcut Approaches to Substance Delivery into the Brain Based on Intranasal Administration Using Nanodelivery Strategies for Insulin. Molecules 25 (21). 10.3390/molecules25215188 PMC7664636 33171799

[B67] Teng C. W. Huang V. Arguelles G. R. Zhou C. Cho S. S. Harmsen S. (2021). Applications of Indocyanine green in Brain Tumor Surgery: Review of Clinical Evidence and Emerging Technologies. Neurosurg. Focus 50 (1), E4. 10.3171/2020.10.focus20782 33386005

[B68] Valdés P. A. Jacobs V. Harris B. T. Wilson B. C. Leblond F. Paulsen K. D. (2015). Quantitative Fluorescence Using 5-aminolevulinic Acid-Induced Protoporphyrin IX Biomarker as a Surgical Adjunct in Low-Grade Glioma Surgery. Jns 123 (3), 771–780. 10.3171/2014.12.jns14391 PMC4646619 26140489

[B69] Viswanathan V. K. Rajaram Manoharan S. R. Subramanian S. Moon A. (2019). Nanotechnology in Spine Surgery: A Current Update and Critical Review of the Literature. World Neurosurg. 123, 142–155. 10.1016/j.wneu.2018.11.035 30447449

[B70] Wang D. Lin B. Ai H. (2014). Theranostic Nanoparticles for Cancer and Cardiovascular Applications. Pharm. Res. 31 (6), 1390–1406. 10.1007/s11095-013-1277-z 24595494

[B71] Wang Y. X. (2011). Superparamagnetic Iron Oxide Based MRI Contrast Agents: Current Status of Clinical Application. Quant Imaging Med. Surg. 1 (1), 35–40. 10.3978/j.issn.2223-4292.2011.08.03 23256052 PMC3496483

[B72] Weber D. C. Lim P. S. Tran S. Walser M. Bolsi A. Kliebsch U. (2020). Proton Therapy for Brain Tumours in the Area of Evidence-Based Medicine. Bjr 93 (1107), 20190237. 10.1259/bjr.20190237 31067074 PMC7066950

[B73] Weidman E. K. Dean K. E. Rivera W. Loftus M. L. Stokes T. W. Min R. J. (2015). MRI Safety: a Report of Current Practice and Advancements in Patient Preparation and Screening. Clin. Imaging 39 (6), 935–937. 10.1016/j.clinimag.2015.09.002 26422769

[B74] Weissleder R. (2006). Molecular Imaging in Cancer. Science 312 (5777), 1168–1171. 10.1126/science.1125949 16728630

[B75] Wu L. C. Zhang Y. Steinberg G. Qu H. Huang S. Cheng M. (2019). A Review of Magnetic Particle Imaging and Perspectives on Neuroimaging. AJNR Am. J. Neuroradiol 40 (2), 206–212. 10.3174/ajnr.a5896 30655254 PMC7028616

[B76] Yao A. D. Cheng D. L. Pan I. Kitamura F. (2020). Deep Learning in Neuroradiology: A Systematic Review of Current Algorithms and Approaches for the New Wave of Imaging Technology. Radiol. Artif. Intelligence 2 (2), e190026. 10.1148/ryai.2020190026 PMC8017426 33937816

[B77] Ze Y. Sheng L. Zhao X. Hong J. Ze X. Yu X. (2014). TiO2 Nanoparticles Induced Hippocampal Neuroinflammation in Mice. PLoS One 9 (3), e92230. 10.1371/journal.pone.0092230 24658543 PMC3962383

[B78] Ze Y. Zheng L. Zhao X. Gui S. Sang X. Su J. (2013). Molecular Mechanism of Titanium Dioxide Nanoparticles-Induced Oxidative Injury in the Brain of Mice. Chemosphere 92 (9), 1183–1189. 10.1016/j.chemosphere.2013.01.094 23466083

[B79] Zhao H., (2016). Tc-HisoDGR as a Potential SPECT Probe for Orthotopic Glioma Detection via Targeting of Integrin Alpha5beta1. Bioconjug. Chem. 27 (5), 1259–1266. 27098436 10.1021/acs.bioconjchem.6b00098

